# How selfish is a thirsty man? A pilot study on comparing sharing behavior with primary and secondary rewards

**DOI:** 10.1371/journal.pone.0201358

**Published:** 2018-08-20

**Authors:** Astrid Kause, Oliver Vitouch, Judith Glück

**Affiliations:** 1 Centre for Decision Research, Leeds University Business School & School of Earth and Environment, The University of Leeds, Leeds, United Kingdom; 2 Harding Center for Risk Literacy, Max Planck Institute for Human Development, Berlin, Germany; 3 Department of Psychology, University of Klagenfurt, Klagenfurt, Austria; Universidad Loyola Andalucia, SPAIN

## Abstract

Human social interactions in daily life involve sharing various types of rewards. Previous research evolving around issues of selfish versus altruistic behavior indicates that when individuals share rewards like money with powerless others, some are purely selfish while a substantial number shares evenly. It is, however, mostly unknown how they share primary rewards like water, compared to secondary rewards like money. We adopt the widely studied Dictator Game for comparing water to be divided among study participants with a monetary reward. We show that thirsty participants share water more often equally with powerless, anonymous others than they do money. This is the case even when they earned both types of rewards in a preceding task. Results indicate that altruistic behavior is more likely to occur when it comes to sharing primary rewards. The ecologically more valid scenario employed in this study provides initial evidence that the concept of a self-interested homo economicus might not apply to everyday social interactions involving rewards other than money.

## Introduction

Daily life circumstances often require sharing various rewards amongst individuals, and those rewards often satisfy primary needs like thirst and hunger [1]. Such primary rewards have been considered different from money as they are strongly associated with situation-specific biological needs. Also, how individuals satisfy needs with primary rewards might depend on old mechanisms evolved over the evolutionary life course [2–4]. Their underlying utility might, thus, differ from money. Money serves as a flexible tool for obtaining rewards that fulfil various needs now or later in time. The occurrence of money and monetary wealth in human evolutionary history is comparably recent and unique to the human species [4]. How individuals behave in and perceive social interactions involving money might thus depend on their social and cultural contexts [4,5].

Does altruistic behavior occur in thirsty humans when water which serves for satisfying a primary, biological need, is the reward to be shared? Notably, most studies exploring sharing behavior have used money as reward to be shared ([6], but see [7]). Little is known about how far their results generalise to other, primary rewards. We investigate how sharing water motivates thirsty individuals to behave more or less fairly towards powerless, anonymous others, compared to money. We also study how recipients’ expectations of amounts received differ, depending on the reward to be shared. We finally explore the extent to which equal sharing behavior occurs when the amount of rewards to be shared is conditional upon performance in a preceding task.

### Theoretical underpinnings of primary and secondary incentives

Different conceptions of the nature of humankind make fundamentally different predictions for primary versus secondary rewards to be shared. The German poet Bertolt Brecht (“Erst kommt das Fressen, dann kommt die Moral” / “A hungry man has no conscience”; ‘The Threepenny Opera‘, [8]) would predict that in real need, most people would share zero. On the other hand, stories like ‘The Bounty Mutiny‘ describe how people share scarce resources like food and water to the point of near-death [9].

Beyond literary sources, divergent predictions can be made on the basis of psychological and economic research. Sharing a primary reward could increase thirsty people’s greediness and motivate them to keep a maximum of the resource for themselves [10]. On the other hand, water to be shared in a situation of need could lead to more altruistic behavior, compared to money. People’s affective empathic feelings for others in a similar situation increase when they themselves, as well as others, are in a physically aroused (‘hot’) state [11]. This might explain why inequality aversion seems to be influencing behavior more when, instead of money, pain is to be divided amongst players [12]. Being better able to empathise with other thirsty people might equally lead to fairer behavior when it comes to sharing a primary reward [7,13]. Also, previous research suggests that in contrast to physical or emotional rewards, money decreases the need for social approval and, thus, also for cooperation with others ('Self-Reliance Theory’ [14]). This implies that selfish behavior might be more prevalent when social interactions involve money. Based on these findings, we tested the hypothesis that thirsty individuals behave more fairly towards others when sharing water as a primary reward compared to money against the assumption that they act more selfishly with water in order to satisfy their own immediate need.

### Earning rewards to be shared with others

The likelihood of observing self-interested behavior substantially increases when participants have *earned* their reward to be shared in some way [15]. It is not clear, however, if such self-regarding behavior generalises to rewards like water which fulfils a primary need. Therefore, we further investigated how sharing behavior of thirsty participants changes, both with respect to water and to money, when they have earned the reward to be shared through individual performance in a preceding task. Based on previous results for money, we expected participants to share less fairly when having earned both water and money.

In order to test these predictions, we use the so-called Dictator Game scenario from the realm of experimental economics, where a ‘proposer’ receives a monetary reward to be shared with a powerless, anonymous recipient [16]. Across studies, a preference for fairness or even altruism shapes behavioral responses in the Dictator Game [6,16,17]. A third of proposers offers 50% or, in rare cases, more, indicating a motivation to act altruistically [6]. This is even the case though proposers have nothing to gain from that behavior but to reduce inequality between players, instead of acting as rational maximizers and keeping everything for themselves.

We assessed water and monetary shares offered by proposers as well as expectations of recipients about how much they will receive [18–20], both for a small amount of water as well as a monetary reward. We used a 2 (shared reward: water versus money; within-subjects) by 2 (kind of stake: earned versus windfall; between-subjects) design. The sample was recruited via the lab of the first author’s institution. In order to induce a primary need for water in advance to the Dictator Game, participants trained on a stationary bike (see Materials and methods).

## Materials and methods

### Sample and procedure

The reported study was approved by the institutional review board of the German Max Planck Institute for Human Development, Berlin. All participants were asked to provide their written informed consent before participating in this study. The methods were carried out in accordance with the relevant guidelines and regulations guidelines for laboratory research of the German Max Planck Society. *N* = 84 participants (50% female, age 18–39 years, *M*_*age*_ = 25.9, *SD* = 3.8, 42 proposers / 42 recipients) were recruited through the lab of the first author’s institution. In the online call for the study, individuals were informed about potential health risks associated with riding a stationary bike. They participated in a short survey assessing their fitness and health impairments. They received 1€ for filling out this survey. Individuals who reported to suffer from chronic or temporary diseases that might have increased the risk of injury from the training session, were excluded from participation. We followed this procedure after consultation of a local physician. Participants agreed to take part in the study by leaving their contact details for recruitment.

In the lab, they were first made thirsty by riding on a stationary bicycle for 25 minutes, similar to a procedure used by van Boven and Loewenstein [21]. In order to ensure the effectiveness of this training, we measured subjective thirst on a 7-point scale from ‘not thirsty at all’ to ‘very thirsty’ before, during and after the sports session. Subjective thirst increased from *M*_*thirst T(first)*_ *=* 3.0 (*SD =* 1.3) to *M*_*thirst T(last)*_ *=* 5.2 (*SD =* 1.4). Proposers tended to keep slightly more water for themselves when they were more thirsty, compared to others (*r*_*thirst T(first)*_ = .12, *p* = .45, *r*^*2*^ = .01; *r*_*thirst T(last)*_ = .19, *p* = .23, *r*^*2*^ = .04). Responders expectations were weakly associated with thirst (*r*_*thirst T(first)*_ = .19, *p* = .24, *r*^*2*^ = .04; *r*_*thirst T(last)*_ = .17, *p* = .30, *r*^*2*^ = .03).

Roles (proposer vs. recipient) were randomly assigned after the training session. 21 pairs participated in the earned condition and 21 pairs in the windfall condition. Proposers then shared both a small amount of water as well as money in randomised order. Both decisions were fully anonymous: the amount of water to be shared was given to proposers in a plastic cup. After the experimenter had left the room, proposers poured the share for the recipient into a second plastic cup. This second cup was then placed in a wooden box so that it was not visible how much proposers offered to recipients and brought to the recipient in a different room. Recipients were first asked to indicate how much money and water they expected to receive from proposers. They then received their water share in the closed box. After the experimenter had left the room, the recipient opened the box and noted down on a questionnaire how much water he had received from the proposer (in ml; in order to facilitate this, he was provided with a measuring cup). After data collection was finished, pairs of participants were matched via the participant numbers noted down on the questionnaires. The money sharing task was performed via a computerized questionnaire. In order to ensure anonymity towards the experimenter [17], shares were paid out via PayPal at the end of the study. Before making their decisions, participants were informed that they would then have to fill out questionnaires for another 30 minutes without a chance to have another drink except for the water to be shared. Demographic variables were assessed after they participated in the game. Before leaving the lab, participants received a show-up fee of €12. During the experiment two participants in the earned condition faced technical problems with the online questionnaire. The answers of one recipient in the windfall condition were missing. These participants were excluded from the analysis.

In advance to the current study, we conducted pilot studies in order to ensure experimental manipulations. In these pilot studies, we measured how much participants wanted to drink after such a training to assess how much water fulfils their immediate need. Based on these results, we determined the amount of water to be shared here. In the earned condition, participants received 100, 150 or 200 ml of water and 5, 7.5 or 10€, depending on the ratio between Watt achieved (i.e. measure of physical exertion) and body weight during minute 15 to 20 of the training on the stationary bike. Ratios were determined after consultation of a local physician. In the windfall condition, participants were randomly assigned to one of the three amounts so that the overall distribution of shares was the same as in the earned condition. Offers did not differ, depending on whether proposers received 100, 150 or 200 ml of water to be shared. This was confirmed in a one-way Analysis of Variance (ANOVA; Welch’s F(2, 15) = .20, *p* = .82; *R*^*2*^_*(adj*.*)*_ = 0.002, Power = 0.84, though these results have to be interpreted with care due to small and unequal group sizes). When they shared 5, 7.5 or 10 Euro, a trend to more selfish shares with larger amount to be shared was observed (Welch’s F(2, 17) = 5.64, *p* = .01, *R*^*2*^_*(adj*.*)*_ = 0.09, Power = 1; for descriptive statistics, we additionally report Bayesian Analyses in the Supplementary Information in S3A–S3D Table). Mean offers per group are displayed in S2 Table. In previous economic games, stake sizes had a low influence on proportion offered [22]). For the analysis, answers were thus pooled across size of incentives.

### Analysis of covariates

In the analysis, we controlled for several covariates. Multilevel linear models predicting shares offered (proposers) or shares expected (responders; Tables 1 and 2) included gender, age, education, frequency of sports per week, smoker (yes/no), time passed since the last drink (in minutes), pulse frequency after the sports session and subjective thirst after the training as main effects. As previous studies employing economic games showed that sharing behavior of monetary rewards was similar across income levels [22] we refrained from measuring need for money at the time point of data collection as additional covariate. All analyses were conducted using the packages ‘Hmisc’ [23], ‘pwr’ [24], and ‘lmerTest’ [25] in R.

**Table 1:** Multilevel regression, predicting Dictator Game offers by proposers.

Variable	b (SE)	t	p
**Intercept**	0.96 (0.28)	3.44	0.001
**Condition (earned vs. windfall)**	0.05 (0.06)	0.81	0.42
**Reward shared (water vs. money)**	-0.38 (0.12)	-3.16	0.003
**Condition x Reward**	0.11 (0.07)	1.54	0.13
**Subjective thirst**	0.01 (0.02)	0.08	0.45
**Time since last drink (minutes)**	0.0003 (0.0002)	1.28	0.24
**Frequency of sport/ week**	-0.07 (0.03)	-2.24	0.03
**Pulse/ minute**	-0.002 (0.001)	-1.69	0.12
**Smoker**	0.02 (0.06)	0.35	0.82
**Order of sharing tasks**	-0.04 (0.05)	-0.97	0.29
**Gender**	0.05 (0.06)	0.81	0.35
**Age**	-0.01 (0.01)	-0.92	0.45
**Education**	-0.01 (0.03)	-0.44	0.68
**AICc = 54.66**			

A simpler model, including only Condition and Reward shared, revealed a similar pattern (AICc_simple_ = -37.57).

**Table 2:** Multilevel regression, predicting expected Dictator Game offers in responders.

Variable	b (SE)	t	p
**Intercept**	0.60 (0.30)	1.98	0.05
**Condition (earned vs. Windfall)**	0.03 (0.69)	0.38	0.71
**Reward shared (water vs. money)**	-0.16 (0.15)	-1.10	0.28
**Condition x Reward**	0.05 (0.09)	0.57	0.58
**Subjective thirst**	0.001 (0.02)	0.10	0.92
**Time since last drink**	0.0001 (0.0002)	0.67	0.51
**Frequency of sport/ week**	-0.002 (0.03)	-0.07	0.94
**Pulse/ minute**	-0.001 (0.001)	-0.81	0.42
**Smoker**	0.10 (0.06)	1.55	0.13
**Order of sharing tasks**	-0.004 (0.05)	-0.09	0.93
**Gender**	-0.03 (0.06)	-0.41	0.68
**Age**	-0.001 (0.01)	-0.27	0.79
**Education**	-0.02 (0.03)	-0.77	0.45
**AICc = 68.98**			

A simple model, including only Condition and Reward shared, revealed a similar pattern (AICc _simple_ = -23.06).

## Results

### Water was shared more generously, compared to money

**Fig 1:**
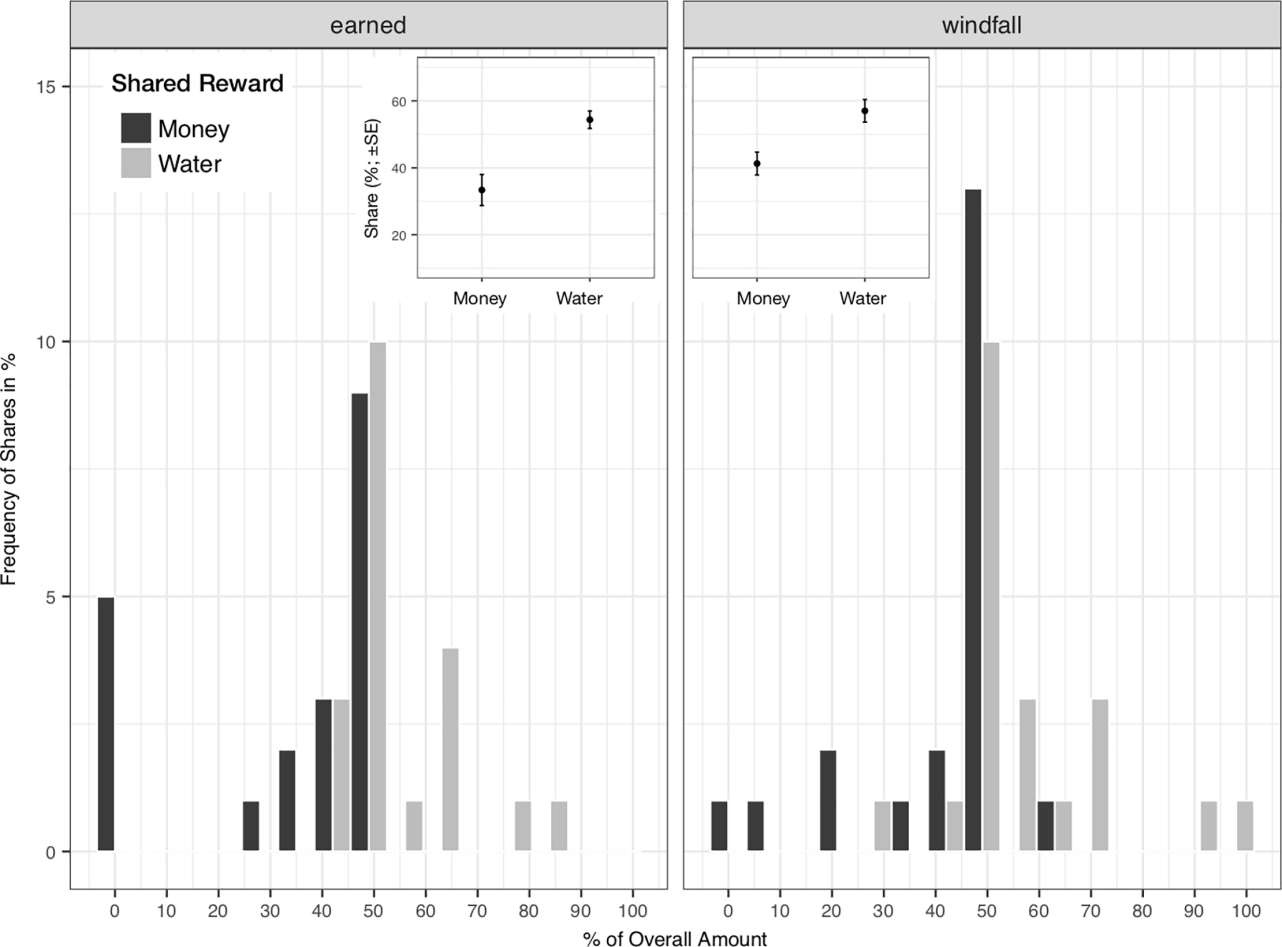
Frequencies of shares in a Dictator Game where thirsty participants shared both water and money (in % of overall shared amount). In the earned-condition (*N* = 20; left panel) the amounts of water and money to be shared were conditional upon performance in a preceding training phase. In the windfall condition (*N* = 21, right panel) proposers received monetary and water amounts independently of their performance. The order of water and money decisions was randomized.

Across experimental groups, proposers offered on average *M*_*water*_ = 56% (*SD* = 14, 95%CI [51, 60]) of the overall amount of water. 41% shared their water equally. 37% gave more than half. No one offered zero. When sharing money, proposers offered on average *M*_*money*_ = 37% (*SD* = 19, 95%CI [31, 43]), while 49% of participants offered half. One person offered more than half and 15% offered nothing (Fig 1). Water offers correlated weakly with monetary offers (*r* = .13, *p* = .40, *r*^*2*^ = 0.2), and differed statistically, with t_*paired*_ (40) = -5.5, *p* < 0.01, Cohen’s *d* = 0.85.

Asking recipients how much they expected to receive from proposers revealed a mean expectation of *M*_*water exp*._ = 46% (*SD* = 13, 95%CI [42, 50]). 75% expected to receive an equal share and 5% expected to receive more than half; and 5% expected to receive nothing. Expected amounts of money were lower, with *M*_*money exp*._ = 36% (*SD* = 21, 95%CI [34, 33]). Here, only 38% expected to receive a fair share, 5% to get more than half and 15% to receive nothing. Money and water expectations did not correlate (*r* = -.08, *p* = .62, *r*^*2*^ = .006). A Wilcoxon-signed rank test confirmed that expected amounts of water were higher than expected amounts of money (U = 81.5, *p* < 0.01, *d =* .38; but see S3D Table).

### Earning rewards to be shared affects monetary offers but not water offers

Water shares were comparably high in the earned and the windfall condition, *M*_*water earned*_ = 54% (*SD* = 12, 95%CI [49, 60]), *M*_*water windfall*_ = 57% (*SD* = 16, 95%CI [50, 64]). With respect to money, participants offered only slightly less when they had earned their stake, *M*_*money earned*_ = 33% (*SD* = 21, 95%CI [28, 43]), and *M*_*money windfall*_ = 41% (*SD* = 16, 95%CI [34, 48]). We predicted offers using a multilevel regression model, including main effects and the interaction of experimental condition (earned versus windfall, between) and shared reward (water versus money, within), and controlled for the covariates described above as main effects. Results confirmed that participants offered less money, compared to water, and made similar offers across conditions. Table 1 reports parameter estimates and model fit indices.

Expectations with respect to water were as high as *M*_*water earned exp*._ = 46% (*SD* = 12, 95%CI [41, 51]) and *M*_*water windfall exp*._ = 46% (*SD* = 15, 95%CI [39, 43]). Recipients expected to receive less money in the earned condition, with *M*_*money earned exp*._ = 32% (*SD* = 20, 95%CI [27, 41]) compared to the windfall condition, with *M*_*money windfall exp*._ = 40% (*SD* = 22, 95%CI [35, 50]). We predicted offers using a multilevel regression model, including main effects and the interaction between experimental condition (earned versus windfall, between) and shared reward (water versus money, within), and controlled for the covariates described above as main effects. Results confirmed that participants expected to receive only slightly more water than money and that expectations were similar across conditions. Table 2 reports parameter estimates and model fit indices.

## Discussion

We investigated whether individuals in need for a primary reward such as water would be more or less willing to give a substantial amount of this reward to others, compared to when they are asked to share money. Our results speak against Bertold Brecht’s dictum of “A hungry man has no conscience” and support the story of the brave Bounty sailors: thirsty individuals in this initial study were more, not less, willing to share a primary reward they have an immediate need for. Results of pilot studies we conducted were similar to the ones reported here (overall *N*_*(proposers pilot)*_ = 43 per experimental group, one group with, one without preceding training session, average amount of water offered *M* = 105 ml, *SD* = 28 [53%]; S1 Table). Interestingly, in the study reported here, to some degree this motivation generalised to sharing money as well.

Even when thirsty participants felt that they had earned their small amount of water they were still willing to share half or more than half of it. In fact, in contrast to money, not a single proposer offered no water whatsoever to his anonymous partner. Our results can potentially be explained by drawing on Van Boven and Loewenstein’s [21] findings that a ‘hot’ physiological state increases empathy, especially when others are in the same state of need. Their willingness to share may not be created by abundance but by personal experiences of being in need.

Furthermore, this response might be more closely related to repeated daily life-experiences and may reflect that altruistic behavior potentially pays back over time as it signals cooperative behavior [26], and is thus adaptive when it comes to satisfying primary needs. Accordingly, the Social Heuristics Hypothesis (SHH) posits that new situations where individual self-control is lower might trigger intuitive, learned, other-regarding responses that paid off previously, instead of more strategic, self-regarding behavior [26–27]: Mild time pressure increases cooperation in economic games, in particular when the situation in the lab is new to study participants [28–29]. Limits of cooperation are reached however under high time pressure as well as when cognitive resources needed for self-control inhibit deliberation about the new situation (‘ego depletion’; [30]).

Similar to light time pressure, studies manipulating ego-depletion via physiological states found that relatively mild hunger seems to increase offers and trust in trust games (statistical effects however were inconclusive, [31]). Interpreting findings in terms of the SHH and available evidence, participants in the reported study may have learned in the past that sharing water when being thirsty is beneficial, paying off through daily repeated interactions. Displayed behaviors may thus represent learned behavioural patterns of social cooperation involving primary reinforcers. Still, the nature of the relationship between ego-depletion and shares is unknown. Then, similar to offers by sleep-deprived dictators [32], strong thirst may decrease shares, possibly implying a U-shaped relationship between cooperation and self-control [30]. Observed differences between rewards may thus be explained by the utility of primary rewards, like water, being non-linear and essentially flat with increasing amounts, while the utility of money increases more linearly. Overall, our findings and conclusions may hold only for this relatively mild and temporary state of need, in contrast to a life-threatening ‘no more water’-situation, as well as situations where individuals are not thirsty at all.

Follow-up studies replicating and extending this initial study should employ a larger sample in order to increase statistical power. The differences in amounts of different types of rewards expected by responders were weaker than the differences between amounts of water and money actually offered by proposers. A larger sample size would allow to further elaborate on our findings.

Such studies may further investigate in how far sharing of primary rewards is influenced by other inter-individual and contextual factors potentially affecting behaviors in daily life, in particular in situations where primary rewards become increasingly scarce [33]. These would include demographic and socio-economic differences. Previous studies revealed that when compared with males, female proposers share money more generously, are expected by others to do so and are more receptive of social context manipulations [19, 34–35]. In contrast to this, amounts offered and expected in this study were similar across males and females. Future research should assess if this extends to primary rewards, both regarding actual offers and expectations [34].

Another factor is the level of need for both rewards. Here, need for money was not controlled for, assuming a linear utility of money rather than context- and time-de-pendency likely associated with a more concave utility function. Further, participants received a show-up fee which might have decreased their need for money. Previous studies investigating how individual differences in need relate to different amounts to be shared produced inconsistent results [36]. These motivate exploring the role of need for money in such comparative studies.

As we employed a double-blind procedure in order to decrease any social influence on monetary decisions [6, 17], money was paid out online, in contrast to water which participants received shortly after. This feature of the experimental design might have shaped responses and motivates both a replication with immediate payout and research on systematically varying time of payout of both rewards. The value of monetary rewards might be more stable whereas it is unclear how individuals perceive water they will receive at a later point in time. The value of water might be either highly context specific and quickly change over time, or remain even more stable compared to money, as current drive states are projected on future needs [21].

More generally, social desirability might have driven behaviors observed [37, 38]. Or, being asked to share two different types of rewards might have made the study pur-pose even more salient and motivated participants to share the second reward more fairly. Both might explain why offers in the money Dictator game were slightly higher compared to previous studies. An additional analysis revealed that money offers were slightly higher when made after the water offer, but this difference was not statistically reliable (*M*_*money 1*_ = 40%, *SD* = .15; *M*_*money 2*_ = 36%, *SD* = .21; t(40) = .7, *p =* .5, *d* = .24). Water offers were similar (*M*_*water 1*_ = 44%, *SD* = .13; *M*_*water 2*_ = 45%, *SD* = .14; t(40) = -0.3, *p* = .80, *d* = .10). Assessing sharing behaviors in a between-design would allow to fully control for order effects, independently of reward shared.

While money-sharing studies have dismissed the idea of homo economicus by showing that humans prefer cooperation and altruism over purely self-interested behavior (e.g., [39]), more naturalistic settings may make an even stronger case for dismissing the concept of egoistic homo economicus. Sharing in daily life frequently involves rewards other than money and findings from research investigating sharing behavior with a primary reward will likely be more ecologically valid than money-sharing tasks, while taking both inter-individual as well as contextual differences into account. Future studies accounting for the above may thus elaborate on our findings. In sum, acting generously and altruistically towards anonymous others might be a frequent motivation when daily life involves primary incentives to be shared.

## Supporting information

S1 Table Overview of pilot studies. (PDF)

S2 Table Mean offers per stake size for water and monetary rewards; in percentage of overall amount. (PDF)

S3 Table Table A. Bayesian correlation between thirst and water offered; proposers, responders. All Bayesian Analyses were conducted using JASP [40]. Table B. Bayesian one-way ANOVA for amount shared (100, 150, 200 ml; 5, 7.5, 10 €), by amount to be shared; proposers. Table C. Correlation of water offers with monetary offers, proposers, and expectations of responders. Table D. Bayesian paired samples T-Test; water offers with monetary offers; proposers; and expectations by responders.
(PDF)
