# Supplementary material for: How selfish is a thirsty man? A pilot study on comparing sharing behavior with primary and secondary rewards
Source: PLoS One. 2018 Aug 20;13(8):e0201358. doi: 10.1371/journal.pone.0201358 (PMC6101360; doi:10.1371/journal.pone.0201358)
Supplement: S1 Table — (PDF) [file pone.0201358.s001.pdf]

How selfish is a thirsty man? A pilot study on comparing sharing behavior with primary and secondary rewards

- S1 Supporting Information -

S1 Table. Overview of pilot studies

Study name and experimenter	Question	Design; sample	Results (in %)
DG with water (I. Gabriel, 2010)	Do participants share water differently, depending on their physical state (thirsty vs. not thirsty)?	2 groups: Group A participated in training, group B did not. Convenience sample University of Klagenfurt; *N* = 43 in each group	Offers of participants participating in training session were higher. $M_{control}$ = 49.41, *SD* = 9.01, $M_{training}$ = 55.93, *SD* = 16.91; t(84) = 2.23, *p* = .03, *d* = .79.
UG with water (C. Hechenbichler, 2011)	How much do participants offer in an UG with water and which offers are accepted?	Proposers/ responders; no experimental variation/ control<br><br>Convenience sample from Carinthian police school; *N* = 30 in each group	Offers are higher than in regular UG. M_{offer} = 62.5, *SD* = 20.5.<br>All offers were accepted, except for 2.
Small unfair offers in DG (A. Kause, 2012)	Do participants accept small, unfair offers? How does water compare to money?	2 groups: Group A receives 15% of share, Group B receives 30% of share. *N* = 51 responders, convenience sample at University of Klagenfurt. Involved deception.	*Water*: 41% accept share as high as 10% of overall amount; 79% accept as high as 30% of overall amount.<br>*Money*: 39% accept 1 out of 10 Euro, 42% accept 2.5 out of 10 Euro.
