# Supplementary material for: How selfish is a thirsty man? A pilot study on comparing sharing behavior with primary and secondary rewards
Source: PLoS One. 2018 Aug 20;13(8):e0201358. doi: 10.1371/journal.pone.0201358 (PMC6101360; doi:10.1371/journal.pone.0201358)
Supplement: S2 Table — (PDF) [file pone.0201358.s002.pdf]

How selfish is a thirsty man? A pilot study on comparing sharing behavior with primary and secondary rewards

- S2 Supporting Information -

S2 Table. Mean offers per stake size for water and monetary rewards; in percentage of overall amount.

	Earned		Baseline	
	Offers	Expectations	Offers	Expectations
100 ml	0.54 (0.04)	0.50 (0)	0.56 (0.05)	0.43 (0.07)
150 ml	0.53 (0.08)	0.29 (0.10)	0.67 (0.12)	0.46 (0.08)
200 ml	0.55 (0.05)	0.50 (0)	0.54 (0.04)	0.47 (0.05)
€5	0.45 (0.03)	0.33 (0.11)	0.53 (0.03)	0.40 (0.31)
€7.5	0.29 (0.10)	0.34 (0.09)	0.44 (0.04)	0.49 (0.06)
€10	0.31 (0.07)	0.31 (0.06)	0.37 (0.05)	0.38 (0.04)
