# Supplementary material for: How selfish is a thirsty man? A pilot study on comparing sharing behavior with primary and secondary rewards
Source: PLoS One. 2018 Aug 20;13(8):e0201358. doi: 10.1371/journal.pone.0201358 (PMC6101360; doi:10.1371/journal.pone.0201358)
Supplement: S3 Table — Table A. Bayesian correlation between thirst and water offered; proposers, responders. All Bayesian Analyses were conducted using JASP [40]. Table B. Bayesian one-way ANOVA for amount shared (100, 150, 200 ml; 5, 7.5, 10 €), by amount to be shared; proposers. Table C. Correlation of water offers with monetary offers, proposers, and expectations of responders. Table D. Bayesian paired samples T-Test; water offers with monetary offers; proposers; and expectations by responders. (PDF) [file pone.0201358.s003.pdf]

How selfish is a thirsty man? A pilot study on comparing sharing behavior with primary and secondary rewards

- S3 Supporting Information -

S3 Table A. Bayesian correlation between thirst and water offered; proposers, responders

	r	Bayes Factor
Proposers		
Thirst; water offered	$r_{thirst\ T(first)} = .12$<br>$r_{thirst\ T(last)} = .19$	.26<br>.39
Responders		
Thirst; water expected	$r_{thirst\ T(first)} = .19$<br>$r_{thirst\ T(last)} = .17$	0.38<br>.33

Note. All Bayesian Analyses were conducted using JASP [40].

S3 Table B. Bayesian one-way ANOVA for amount shared (100, 150, 200 ml; 5, 7.5, 10 €), by amount to be shared; proposers

Models	p(M)	p(M|data)	Bayes $Factor_M$	Bayes $Factor_{01}$	error %
Water					
Null model	0.5	0.80	3.91	1	
Amount shared	0.5	0.20	0.26	3.91	0.04
Money					
Null model	0.5	0.57	1.35	1	
Amount shared	0.5	0.43	0.74	0.74	0.02

S3 Table C. Correlation of water offers with monetary offers, proposers, and expectations of responders

	r	Bayes Factor
Proposers		
Water offers, monetary offers	.14	0.27
Responders		
Expectations water offers, monetary offers	-.08	0.22

S3 Table D. Bayesian paired samples T-Test; water offers with monetary offers; proposers; and expectations by responders

	Bayes Factor	Error (%)
Proposers		
Water offers, monetary offers	6540	6.06E-10
Responders		
Expectations water offers, monetary offers	2.28	1.32E-07
